# A Role for Interleukin-1 Alpha in the 1,25 Dihydroxyvitamin D_3_ Response in Mammary Epithelial Cells

**DOI:** 10.1371/journal.pone.0081367

**Published:** 2013-11-07

**Authors:** Sophia L. Maund, Lihong Shi, Scott D. Cramer

**Affiliations:** 1 Department of Cancer Biology, Wake Forest University School of Medicine, Winston-Salem, North Carolina, United States of America; 2 Department of Pharmacology, University of Colorado Anschutz Medical Campus, Aurora, Colorado, United States of America; University of Tennessee, United States of America

## Abstract

Breast cancer is the most common non-cutaneous malignancy in American women, and better preventative strategies are needed. Epidemiological and laboratory studies point to vitamin D_3_ as a promising chemopreventative agent for breast cancer. Vitamin D_3_ metabolites induce anti-proliferative effects in breast cancer cells *in vitro* and *in vivo*, but few studies have investigated their effects in normal mammary epithelial cells. We hypothesized that 1,25(OH)_2_D_3_, the metabolically active form of vitamin D_3_, is growth suppressive in normal mouse mammary epithelial cells. In addition, we have previously established a role for the cytokine interleukin-1 alpha (IL1α) in the anti-proliferative effects of 1,25(OH)_2_D_3_ in normal prostate cells, and so we hypothesized that IL1α is involved in the 1,25(OH)_2_D_3_ response in mammary cells. Evaluation of cell viability, clonogenicity, senescence, and induction of cell cycle regulators p21 and p27 supported an anti-proliferative role for 1,25(OH)_2_D_3_ in mammary epithelial cells. Furthermore, 1,25(OH)_2_D_3_ increased the intracellular expression of IL1α, which was necessary for the anti-proliferative effects of 1,25(OH)_2_D_3_ in mammary cells. Together, these findings support the chemopreventative potential of vitamin D_3_ in the mammary gland and present a role for IL1α in regulation of mammary cell proliferation by 1,25(OH)_2_D_3_.

## Introduction

Epidemiological and laboratory studies point to vitamin D_3_ as a promising chemopreventative agent for breast cancer [1-7]. Rigorous clinical studies are lacking, but increasing evidence highlights the importance of vitamin D_3_ in maintaining breast health [8-11]. Low serum 25(OH)D_3_ concentrations are correlated with an increased risk for breast cancer [3], and suboptimal serum 25(OH)D_3_ levels are associated with more aggressive breast tumors, worse prognostic markers, and a higher risk for breast cancer recurrence [12]. These findings support reports of increased breast cancer risk and decreased survival in patients deficient in vitamin D_3_, and they warrant further investigations into the specific contributions of vitamin D_3_ to breast health.

Mammary epithelial cells endogenously express 1 alpha-hydroxylase (1α-OHase, encoded by CYP27B1) and can therefore generate 1,25 dihydroxyvitamin D_3_ (1,25(OH)_2_D_3_), the biologically active form of vitamin D_3_, from 25(OH)D_3_ in autocrine and paracrine manners [13], which supports a role for 1,25(OH)_2_D_3_ in mammary gland function and homeostasis [14]. The vitamin D receptor (VDR) is expressed in all cells of the mammary tissue and it is actively regulated during puberty and pregnancy; its levels increase 100-fold throughout lactation [15]. VDR-knockout mice exhibit excessive mammary epithelial proliferation and impaired apoptosis [15,16], and breast tumors with higher VDR expression are correlated with better patient prognosis [17]. Furthermore, CYP27B1 (which encodes the 1,25(OH)_2_D_3_ activating enzyme 1α-OHase) expression is slightly lower in invasive breast tumors, while CYP24A1 (which encodes the 1,25(OH)_2_D_3_ inactivating enzyme 24-hydroxylase) levels are increased in tumors compared to benign lesions [18]. These studies suggest that breast cancer is associated with deregulation of vitamin D_3_ signaling. These and other *in vitro* and *in vivo* studies support the protective effects of 1,25 (OH)_2_D_3_ against breast cancer development and progression [1,19,20].

We previously reported a novel role for interleukin-1 alpha (IL1α) in the anti-proliferative effects of 1,25(OH)_2_D_3_ in the prostate progenitor/stem cell (PrP/SC) [21]. IL1α is a multi-functional cytokine that is classically characterized as pro-inflammatory, but it has more recently been reported to regulate cell proliferation, differentiation, and senescence in a cell-type-dependent manner [22-38]. Furthermore, while secreted IL1α and membrane-bound IL1α contribute to inflammation and immune responses, intracellular IL1α is hypothesized to exert anti-proliferative and pro-differentiation effects [39]. IL1α is one of only two interleukins that contain a nuclear localization sequence [40]. The precise nuclear role(s) of IL1α is still unclear, but studies suggest that it can impact transcription through interaction with RNA processing machinery, histone acetyltransferases, and transcription factors [41-44]. 

IL1α expression or activity has not previously been studied in benign mammary cells, neither alone nor in response to 1,25(OH)_2_D_3_. The effects of IL1α and 1,25(OH)_2_D_3_ in mammary cells is relevant to the study of vitamin D_3_ in the chemopreventative setting. Here, we report that 1,25(OH)_2_D_3_ induces IL1α expression in normal mouse mammary epithelial cells (MMECs), and that IL1α contributes to the anti-proliferative effects of 1,25(OH)_2_D_3_ in these cells.

## Materials and Methods

### Ethics statement

This study was approved by the Wake Forest University School of Medicine Animal Care and Use Committee. The method of sacrifice was carbon dioxide inhalation followed by cervical dislocation. 

### Isolation and culture of mouse mammary epithelial cells

Normal MMECs were isolated from C57BL/6; 129/SVEV mice as described in detail in [45]. The isolated cells were primarily basal epithelial cells, and vitamin D receptor expression was confirmed by reverse-transcriptase PCR (data not shown). Cells were cultured in complete DMEM/F12 as described in [46], and experiments were performed between passages 20-30. 

### Antibodies and reagents

Antibodies: p21 and p27, Cell Signaling Technology (Danvers, MA, USA); IL1α and IL1RI, Santa Cruz Biotechnology Inc. (Santa Cruz, CA, USA); β-actin, Sigma Aldrich (St. Louis, MO, USA); AlexaFluor 488 anti-Rabbit, Invitrogen (Carlsbad, CA, USA). Reagents: 1,25(OH)_2_D_3_, BIOMOL international (Plymouth Meeting, PA, USA). When BIOMOL was integrated into Enzo Life Sciences, 1,25(OH)_2_D_3_ was purchased from Sigma Aldrich.

### Immunoblotting

Procedures for immunoblotting protein lysates from cells grown in monolayer is described in detail elsewhere [46]. Immunoblot experiments were repeated at least once and densitometry was performed using ImageJ software.

### Growth assays

Trypan blue exclusion assays were performed as described in [47]. Briefly, cells were plated at 1 x 10^4^ cells per 35 mm culture dish (n = 3, 4, or 5 replicate preparations). The medium was replaced with experimental media 24 hrs after plating. When control cells reached 90% confluency, cells were collected, trypan blue was administered, and total and non-viable cells were counted. The mean number of viable cells per dish and percentages of viable cells were calculated and statistical significance was verified using ANOVA (critical value = 0.05) with post-hoc analysis by Fisher’s LSD test using the statistical software package NCSS 6.0.22.

### Clonogenic assays

Clonogenic assays were performed as described in Barclay et al. [48]. Briefly, cells were plated at 250 cells per 60 mm culture dish (n = 3 replicate preparations) in experimental or control medium. Cells were fixed and stained with 0.1% crystal violet in 95% ethanol after 9 days. Colonies (defined as >50 cells) were counted and the total areas were calculated in pixels using Adobe Photoshop Elements. Statistical determinations were calculated by ANOVA with post-hoc analysis by Fisher’s LSD test using the statistical software package NCSS 6.0.22.

### Quantitative real-time PCR analysis (qPCR)

RNA was isolated from MMECs treated in triplicate with vehicle (0.1% ethanol) or 100 nM 1,25(OH)_2_D_3_ for 24 hrs, quantified and converted to cDNA using reverse transcriptase, and diluted 1:10 in H_2_O. qPCR was performed using Bio-Rad iQ SYBR green super-mix (Bio-Rad, Hercules, CA, USA). The results were analyzed using delta-delta Ct calculations, normalized to Gapdh expression levels, and further normalized to the gene expression levels under vehicle control-treated conditions (error bars show standard deviations). Statistical significance was determined by T-test (critical value = 0.05), n = 3 replicate preparations. IL1α qPCR primers were from SABiosciences (Frederick, MD, USA). Additional qPCR primer sequences are as follows: Cdkn1a f-GACAAGAGGCCCAGTACTTCC, r-CAGACACCAGAGTGCAAGAC; Cdkn1b f-GGACTTGGAGAAGCACTGC, r-CACCTCCTGCCACTCGTATC; Cyp24a1 f-GAAGATGTGAGGAATATGCCCTATTT, r-CCGAGTTGTGAATGGCACACT; Gapdh f-TGCGACTTCAACAGCAACTC, r-GCCTCTCTTGCTCAGTGTCC.

### shRNA targeting

shRNA vectors were generated as described in Sui and Shi [49]. The IL1α target site was GGTAGTGAGACCGACCTCATT. After infection with ecotropic virus, single cell clones were isolated using cloning cylinders, and IL1α protein expression was evaluated by Western blot after 24 hr treatments with 100 nM 1,25(OH)_2_D_3_ or 0.1% ethanol. Viral infection efficiency was validated by a positive GFP signal encoded by the virus. 

### Immunofluorescence

Immunofluorescence was performed as described in [50]. Fluorescent signal images were captured using a Nikon DXM1200F digital camera on a Nikon Eclipse 50i microscope with an EXFO X-Cite 120 Fluorescence Illumination System.

### Senescence-associated beta-galactosidase (SA-β-gal) assay

SA-β-gal activity was evaluated as described in Axanova et al.[51].

## Results

### 1,25(OH)_2_D_3_ inhibits mammary cell growth and induces p21 and p27

**Figure 1:**
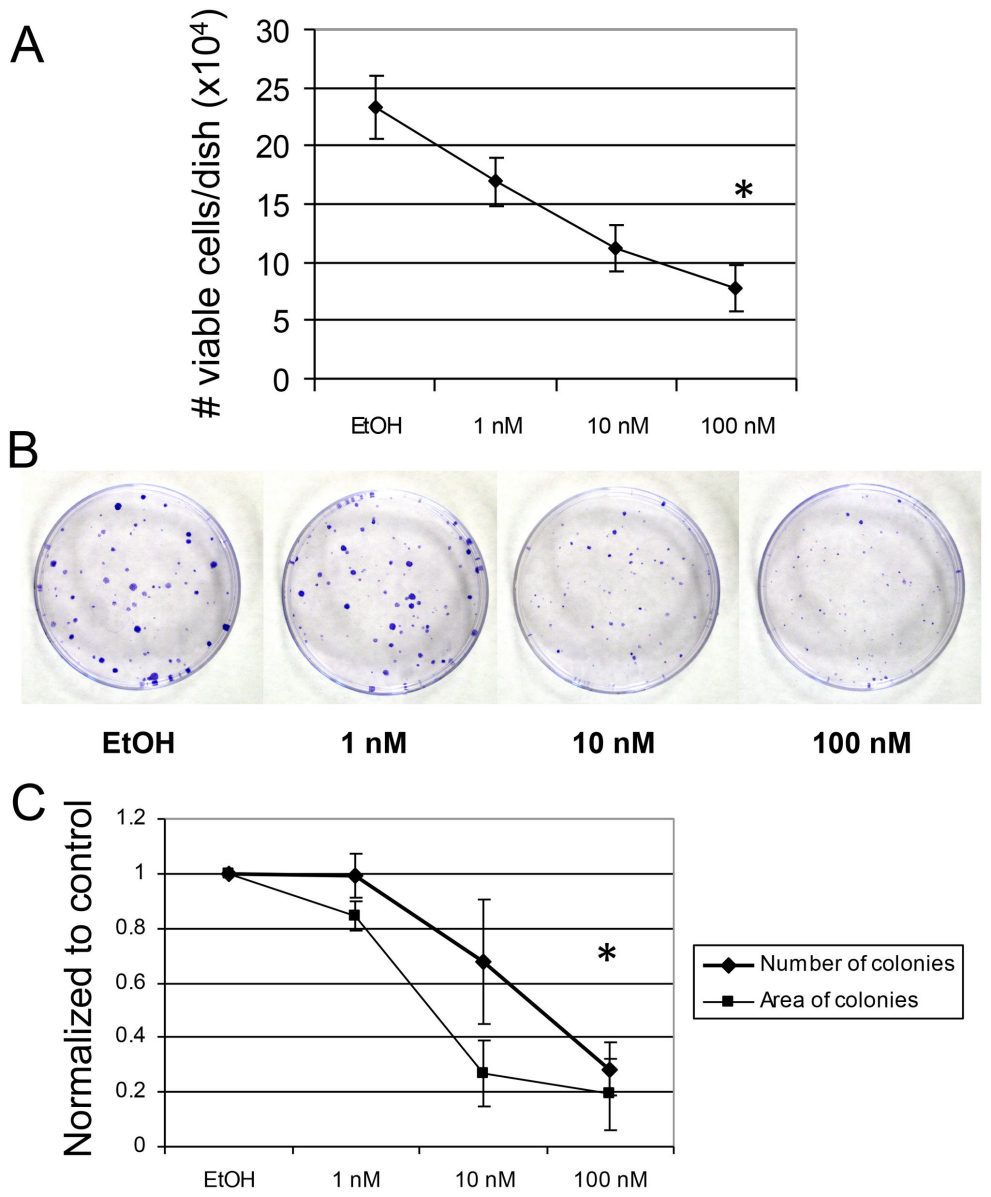
1,25(OH)_2_D_3_ inhibits MMEC growth. (A) MMECs were treated with the indicated doses of 1,25(OH)_2_D_3_ or 0.1% vehicle control (EtOH) for 48 hours. Viable cells were counted according to trypan blue exclusion. * = p < 0.05. (B) Representative images from clonogenic assays, quantified in (C). * = p < 0.05.

**Figure 2:**
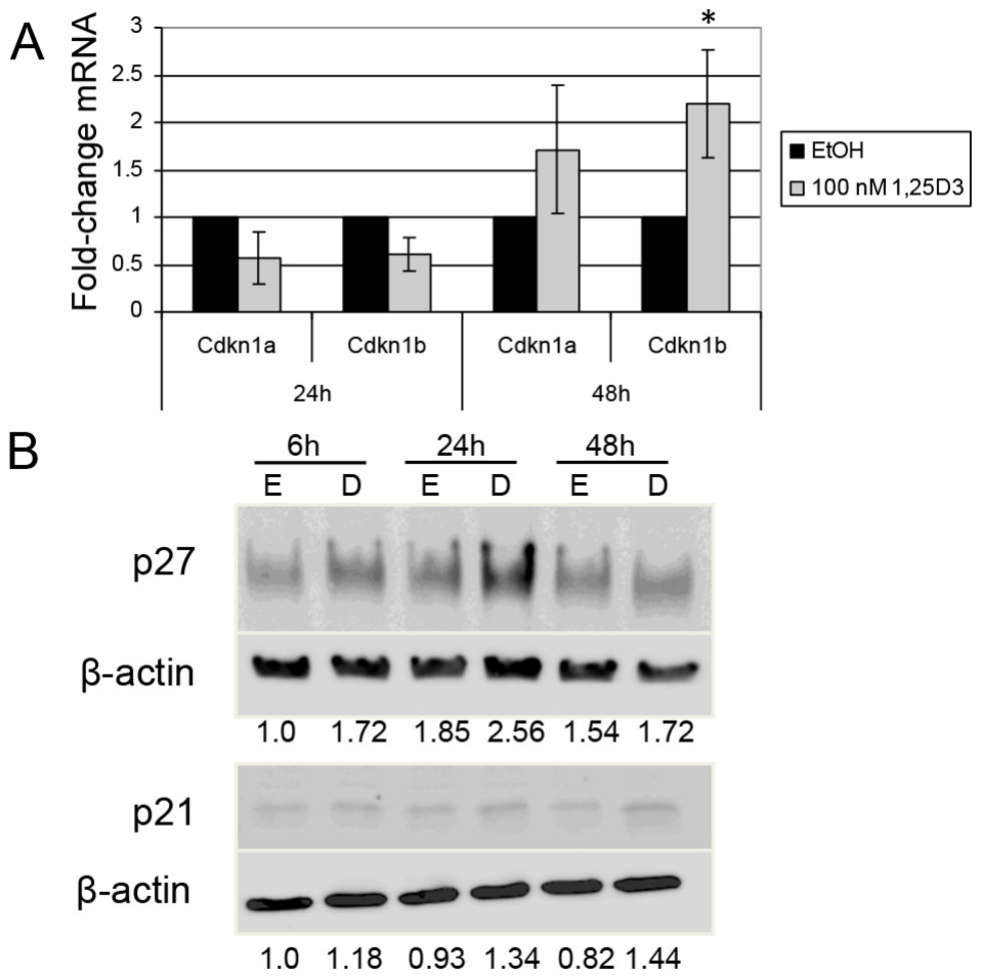
1,25(OH)_2_D_3_ induces p27 in MMECs. (A) qPCR indicated induction of Cdkn1b mRNA at 48 hrs of 100 nM 1,25(OH)_2_D_3_ (1,25D3, * = p < 0.05) and a non-significant trend toward induction of Cdkn1a at 48 hrs. (B) Induction of p27 protein was detected at 6 and 24 hrs of treatment with 100 nM 1,25(OH)_2_D_3_ (D) compared to EtOH control (E). The p21 antibody signal was weak.

1,25(OH)_2_D_3_ has been shown to inhibit growth of benign and malignant breast epithelial cells [20,52,53]. We previously isolated normal MMECs from B1/6; 129/SVEV mice. A trypan blue exclusion assay revealed that 1,25(OH)_2_D_3_ elicited dose-dependent growth inhibition of MMECs at 48 hrs (Figure **1A**). 1,25(OH)_2_D_3_ also inhibited clonogenic growth of MMECs (Figure **1B and C**). These results verify the growth-suppressive effects of 1,25(OH)_2_D_3_ in normal MMECs. p21 and p27, encoded respectively by Cdkn1a and Cdkn1b, are common downstream targets of 1,25(OH)_2_D_3_ that contribute to cell cycle arrest in breast cancer cells [53,54]. qPCR showed a non-significant trend toward induction of Cdkn1a and significant induction of Cdkn1b mRNA after 48 hrs of 1,25(OH)_2_D_3_ in MMECs (Figure **2A**). However, protein levels of p27 increased by 6 and 24 hours of 1,25(OH)_2_D_3_, while p21 protein induction was minimal (Figure **2B**, Figure **1**). These results were consistent in three experimental replicates; we speculate that the p21 expression is low in these cells, giving a generally weak signal by immunoblot which made quantification difficult. p21 and p27 can be regulated post-transcriptionally and post-translationally, so the disconnect between mRNA and protein expression patterns is likely due to unknown secondary regulation that may occur at earlier (6-24 hrs) versus later (48 hrs) time points [55-58]. Regardless, 1,25(OH)_2_D_3_ induced significant growth inhibition of MMECs that was consistent with p27 protein induction.

### 1,25(OH)_2_D_3_ induces senescence in mammary epithelial cells

**Figure 3:**
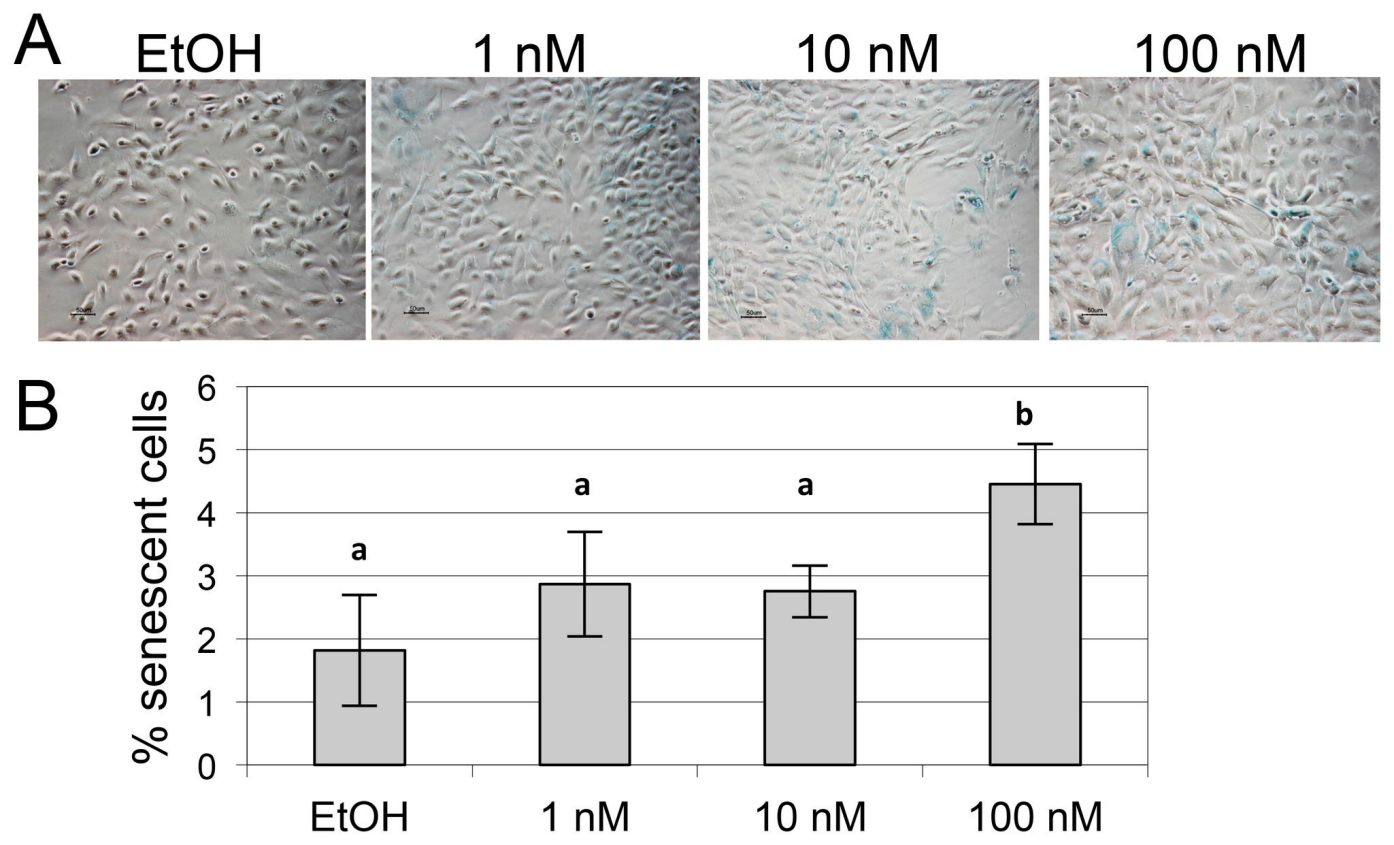
1,25(OH)_2_D_3_ induces senescence of MMECs. (A) Representative images from the SA-β-gal assays quantified in (B). 100 nM 1,25(OH)_2_D_3_ significantly induced senescence compared to the control treatment (EtOH). Bars labeled “a” or “b” are statistically significantly different from each other according to ANOVA and post-hoc Fisher’s LSD test (n = 3 replicates, ~160 cells quantified in each of 10 fields of view per replicate, critical value = 0.05).

We previously reported that 1,25(OH)_2_D_3_ induces senescence in prostate cancer cell lines as well as in normal PrP/SC in dose-dependent manners [21,51]. We hypothesized that 1,25(OH)_2_D_3_ can induce senescence in mammary epithelial cells as well. We performed a senescence-associated beta galactosidase (SA-β-gal) assay in MMECs treated with vehicle control (0.1% ethanol) or increasing doses of 1,25(OH)_2_D_3_ every 48 hours for 96 hours. Senescent cells are characterized by an enlarged, flattened morphology and SA-β-gal expression. We found that 100 nM 1,25(OH)_2_D_3_ significantly induced MMEC senescence (Figure **3**), indicating that induction of senescence by 1,25(OH)_2_D_3_ is not a prostate-specific effect. Induction of senescence may be considered one mode of 1,25(OH)_2_D_3_-mediated growth inhibition in both mammary and prostate cells.

### 1,25(OH)_2_D_3_ induces IL1α in mammary epithelial cells

**Figure 4:**
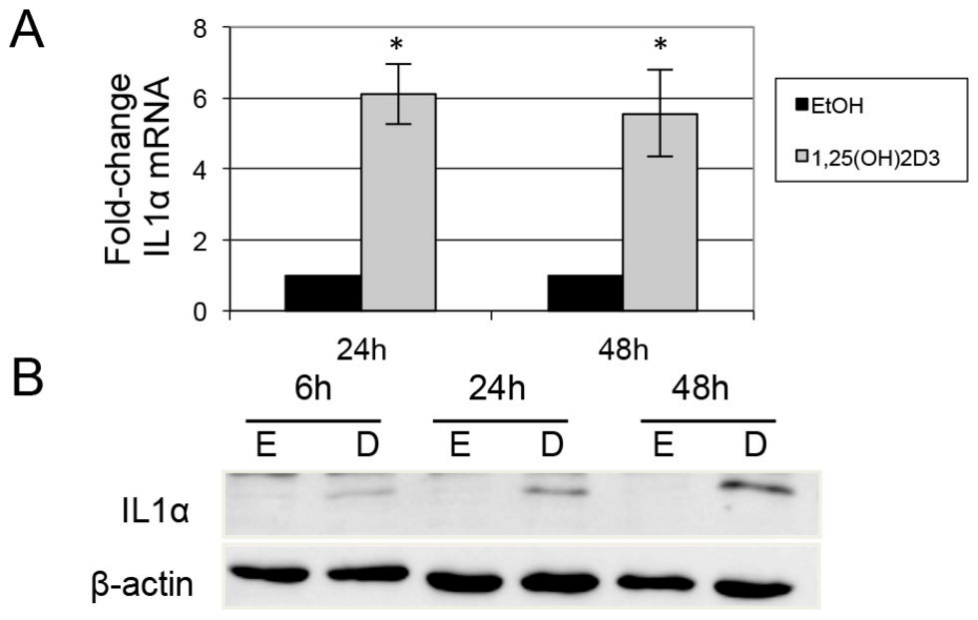
1,25(OH)_2_D_3_ induces IL1α mRNA and protein in MMECs. (A) qPCR revealed a 5 to 6-fold induction of IL1α mRNA by 100 nM 1,25(OH)_2_D_3_. * = p < 0.001. (B) 100 nM 1,25(OH)_2_D_3_ (D) induced IL1α protein at 6, 24, and 48 hours. Very little IL1α was present in the cells treated with ethanol control (E).

We previously identified IL1α as a novel, prominent downstream signaling target of 1,25(OH)_2_D_3_ in mouse PrP/SC. To test whether IL1α is a target of 1,25(OH)_2_D_3_ in mammary cells as well, we investigated its regulation in MMECs in response to 1,25(OH)_2_D_3_. qPCR data showed a 6-fold increase in IL1α mRNA by 24 hrs of exposure to 1,25(OH)_2_D_3_, which was sustained at 48 hr (Figure **4A**). Immunoblot blot analysis revealed induction of IL1α protein at 6 hrs and more robust induction at 24 and 48 hrs. (Figure **4B**, Figure **2**). IL1α protein was virtually undetectable in the absence of 1,25(OH)_2_D_3_. We previously identified a putative vitamin D response element (VDRE) in the promoter region of IL1α that aligns with established VDREs in other targets of 1,25(OH)_2_D_3_ [21], so it is possible that IL1α is a direct transcriptional target of 1,25(OH)_2_D_3_. Together with our previous report we identify IL1α as a downstream target of 1,25(OH)_2_D_3_ in both mammary and prostate epithelial cells [21].

### Cellular localization of IL1α and IL1R1 in mammary epithelial cells

**Figure 5:**
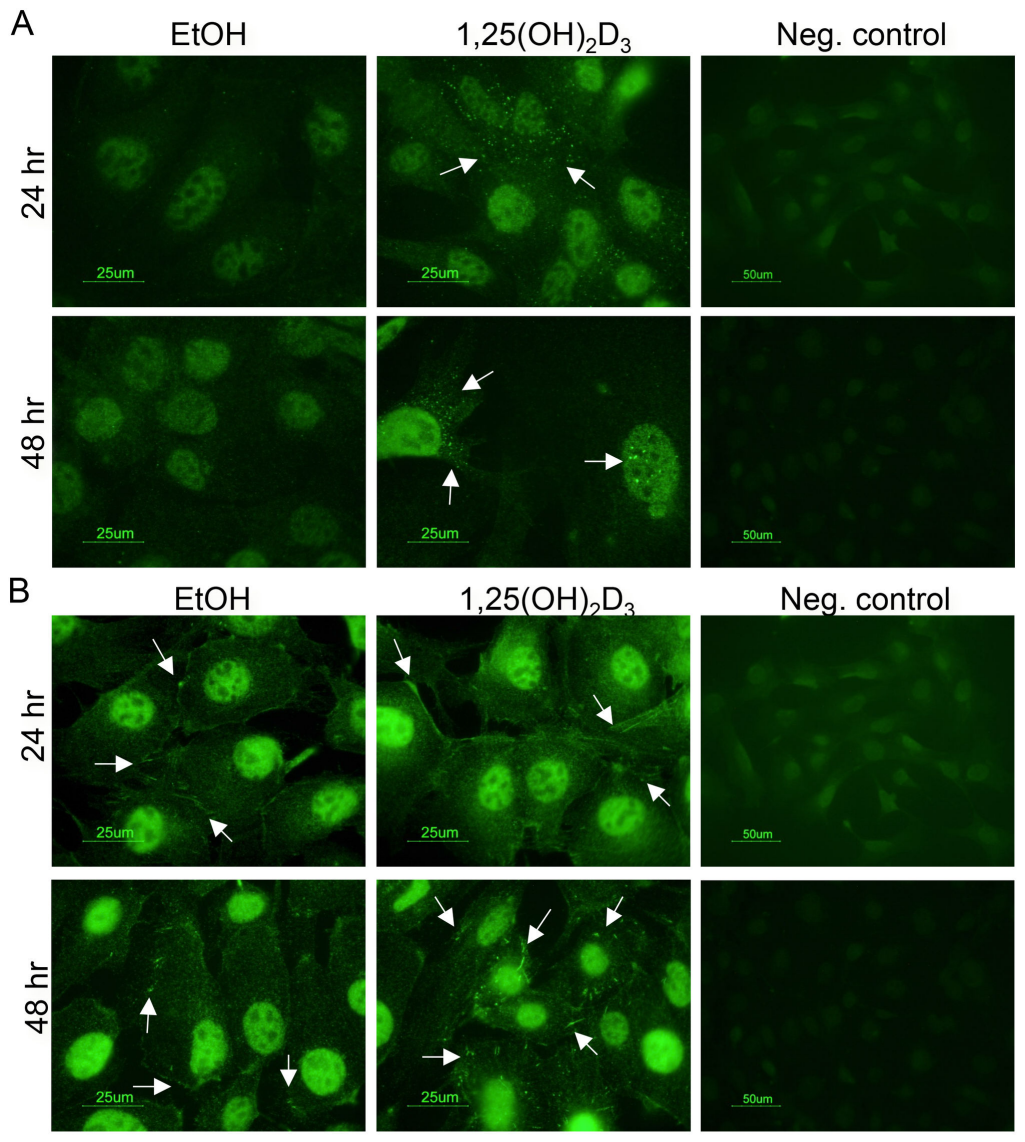
Cellular localization of IL1α and IL1R1 in MMECs. (A) Punctate IL1α signals (arrows) were detected in the nuclear and cytoplasmic compartments of MMECs upon 24 and 48 hr treatments with 100 nM 1,25(OH)_2_D_3_. IL1α was undetected in 0.1% vehicle control-treated cells (EtOH) and under negative control conditions (no primary antibody). (B) IL1RI signal was detected at the edges of the cell membranes at 24 and 48 hours in MMECs treated with 0.1% vehicle control (EtOH, arrows). IL1RI was detected both at the edges of the cells and within the cytoplasmic compartments after treatment with 100 nM 1,25(OH)_2_D_3_ for 48 hrs (arrows). No signal was detected under negative control conditions.

IL1α is rarely secreted from epithelial cells [21,59], but it can be tethered to the cell membrane or shuttled to the nucleus via its nuclear localization sequence. This distinction is important because the cellular localization of IL1α likely determines its downstream effects [39]. We used immunofluorescence to visualize the cellular localization of IL1α in MMECs treated with 100 nM 1,25(OH)_2_D_3_ or vehicle control (0.1% ethanol) for 24 and 48 hrs. IL1α was localized to the nuclear and cytoplasmic compartments of MMECs treated with 100 nM 1,25(OH)_2_D_3_ for both 24 and 48 hours (Figure **5A**, arrows). As expected, IL1α signal was not detected in the ethanol control-treated cells, nor was it detected under negative control conditions (no primary antibody, Figure **5A**). The localization of 1,25(OH)_2_D_3_-induced IL1α mirrors that in the prostate stem cell [21] and suggests an intracrine function for IL1α in response to 1,25(OH)_2_D_3_. 

Previous reports have demonstrated cellular uptake and intracellular interaction of IL1R1 with IL1α [60,61]. In control-treated MMECs, we observed IL1RI signal at the edges of the cell membranes at both 24 and 48 hours (Figure **5B**, arrows). Interestingly, in MMECs treated with 100 nM 1,25(OH)_2_D_3_, IL1RI appeared to be localized both at the edges of the membrane and in the cytoplasmic compartment, especially at 48 hours (Figure **5B**, arrows). A similar pattern was observed in the prostate stem cell [21], which contributes to speculation that IL1α may interact with IL1RI and promote intracellular translocation. Further studies will be necessary to investigate a possible intracellular role of the IL1R1/ IL1α complex. To our knowledge, this is the first report showing endogenous expression patterns of IL1α and IL1RI in mammary epithelial cells, particularly in response to 1,25(OH)_2_D_3_.

### IL1α is necessary for the anti-proliferative effects of 1,25(OH)_2_D_3_ in MMECs

**Figure 6:**
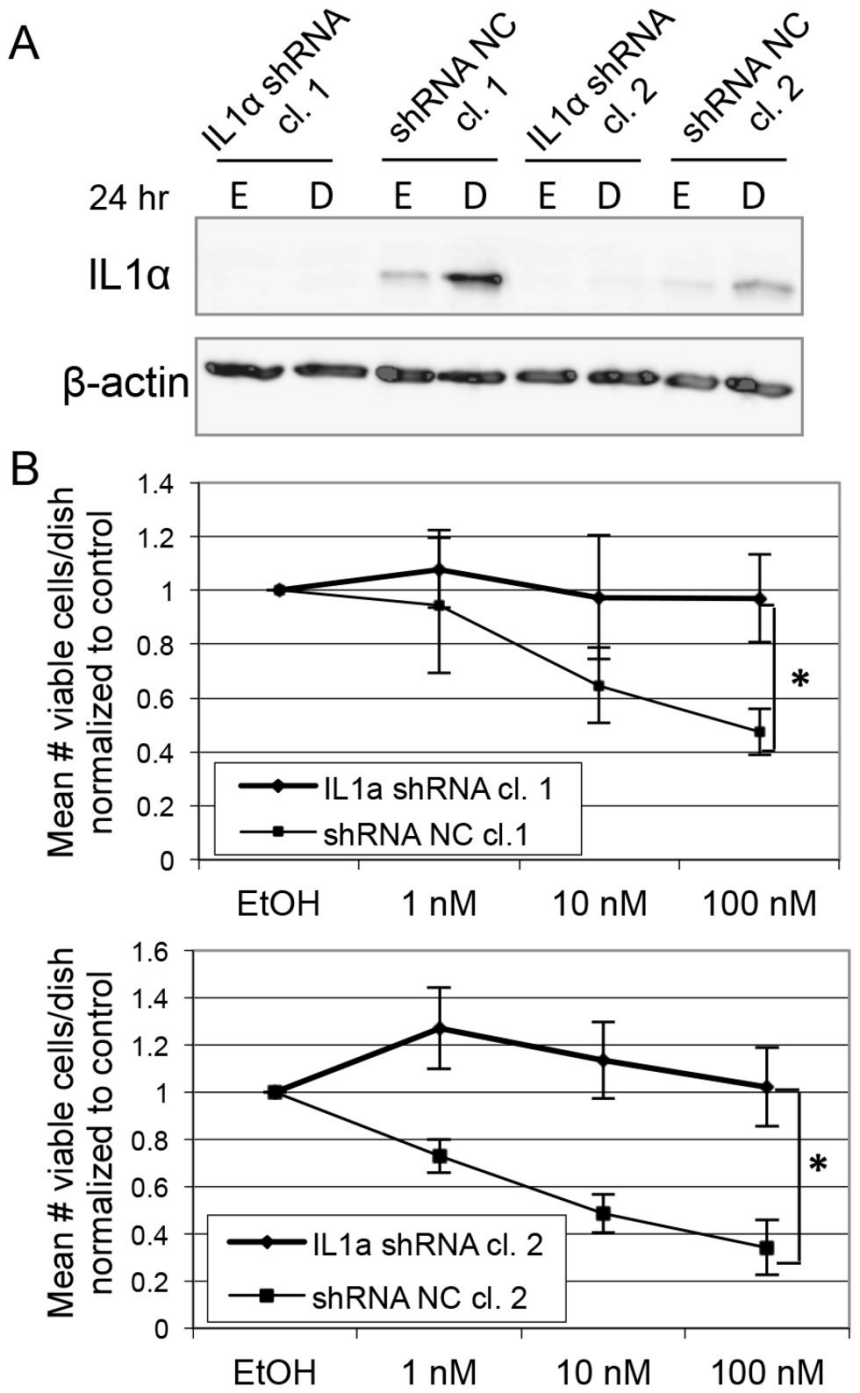
IL1α mediates the anti-proliferative effects of 1,25(OH)_2_D_3_ in MMECs. (A) Western blot for IL1α expression in MMEC clones (cl.) infected with IL1α shRNA or negative control shRNA (shRNA NC). E = 0.1% ethanol, D = 100 nM 1,25(OH)_2_D_3_. (B) 48 hr trypan blue exclusion assays in MMEC clones infected with IL1α shRNA or control shRNA. * = p < 0.05.

Because IL1α is highly upregulated by 1,25(OH)_2_D_3_ in MMECs and is necessary for 1,25(OH)_2_D_3_-mediated growth inhibition of prostate stem cells, we hypothesized that IL1α contributes to the anti-proliferative effects of 1,25(OH)_2_D_3_ in MMECs. We infected MMECs with previously validated lentiviral shRNA vectors targeting IL1α or a scrambled control sequence. We selected clonal populations of shRNA-infected MMECs that achieved complete and stable knock down of IL1α. We validated the knock down of IL1α by Western blot in cells treated for 24 hrs with 0.1% ethanol control or 100 nM 1,25(OH)_2_D_3_. IL1α protein expression was induced by 1,25(OH)_2_D_3_ in the control-infected MMECs (shRNA NC), and it was absent in the IL1α shRNA-infected MMECs (Figure **6A**). 

We next tested the effects of 1,25(OH)_2_D_3_ on cell growth and viability in the control and IL1α shRNA-infected MMECs. MMECs infected with control shRNA were growth-inhibited by 1,25(OH)_2_D_3_ in a dose-dependent manner as expected according to a 48 hr trypan blue exclusion assay (Figure **6B**). However, IL1α shRNA-infected MMECs were resistant to the growth-inhibitory effects of 1,25(OH)_2_D_3_ (Figure **6B**). This suggests that IL1α is necessary for the anti-proliferative effects of 1,25(OH)_2_D_3_ in MMECs. To verify that the IL1α knock-down clones otherwise remained responsive to 1,25(OH)_2_D_3_, we performed qPCR for Cyp24a1, a well-described universal target of 1,25(OH)_2_D_3_ (Figure **3**). Cyp24a1 mRNA levels were robustly induced by 1,25(OH)_2_D_3_ in all MMEC clones, suggesting that 1,25(OH)_2_D_3_ signaling remained intact and that the anti-proliferative effects of 1,25(OH)_2_D_3_ are in fact dependent on the presence of IL1α. 

## Discussion

While the anti-proliferative and pro-differentiation effects of 1,25(OH)_2_D_3_ have been characterized in breast cancer cells, few studies have focused on the effects of 1,25(OH)_2_D_3_ in normal mammary cells. Such studies are critical for gaining a better understanding of a mechanistic basis for chemoprevention by vitamin D_3_. Here we have shown that 1,25(OH)_2_D_3_ reduces normal MMEC proliferation and induces senescence. Furthermore, IL1α is necessary for the anti-proliferative effects of 1,25(OH)_2_D_3_ in MMECs, and it likely acts in cytoplasmic and/or nuclear compartments.

Others have begun to interrogate the genomic effects of 1,25(OH)_2_D_3_ in normal and malignant human mammary cells and in mouse mammary tumor cells, but we are the first to report IL1α as a target of 1,25(OH)_2_D_3_ signaling in mammary cells [19,62]. Microarray studies have revealed that 1,25(OH)_2_D_3_ regulates a wide variety of genes involved in innate immunity, differentiation, metabolism, and extracellular matrix remodeling. It appears that specific 1,25(OH)_2_D_3_ target genes vary with the cell lines, model systems, and microarray platforms used. Additional microarray analyses from normal and malignant breast cells and subsequent experimental validation of potential 1,25(OH)_2_D_3_ targets will help shed light on the mechanistic actions of 1,25(OH)_2_D_3_ in the breast, informing the use of vitamin D_3_ in preventative and clinical settings. While most studies on the mechanistic action of vitamin D_3_ focus on the metabolic intermediate 1,25(OH)_2_D_3_, some data from other tissues suggest that alternative metabolic intermediates are generated *in vivo* [63]. The generation of these intermediates may also be important for vitamin D_3_ action. However, the role of these intermediates in any biological process, including growth inhibition of mammary cells, is as yet undefined. 

Some of the common downstream targets of 1,25(OH)_2_D_3_ signaling in breast cancer cells include BRCA1, p21, p53, cMYC, E-Cadherin, and Cyclin D1, which contribute to cell cycle arrest, differentiation, and, at times, apoptosis [20,52,64,65]. 1,25(OH)_2_D_3_ has also been reported to inhibit matrix metalloproteinase (MMP) and urokinase-type plamsinogen activator (uPA) production and enhance tissue inhibitors of matrix metalloproteinases (TIMPs), which may help impede breast cancer cell invasion and metastasis [66,67]. Induction of p27 by1,25(OH)_2_D_3_ may contribute to its anti-proliferative effects in MMECs. p27 is a well-characterized mediator of cell cycle arrest, and it has also been implicated in induction of senescence [68]. Interestingly, IL1α is also implicated in senescence in some cell types including HUVECs, prostate cells, and fibroblasts [35,36,38]. However, induction of senescence by 1,25(OH)_2_D_3_ persisted upon knockdown of IL1α, suggesting that other signaling targets, such as p27, are likely mediate 1,25(OH)_2_D_3_-induced senescence in MMECs (Figure **4**). Reports in prostate cancer models suggest that reduction or inhibition of p27 blocks induction of senescence [68,69], and investigations into the precise roles of p27 and IL1α in senescence in multiple cell types are ongoing. 

IL1α activity has previously been reported in breast cancer cells outside of the context of vitamin D_3_. In 1988, recombinant IL1α was first reported to inhibit growth of estrogen-dependent breast cancer cell lines MDA-MB-415 and MCF-7, but not that of hormone-independent breast cancer cell lines (HS-578-T and MDA-231) [27,70]. Later, IL1α was shown to inhibit estrogen-mediated growth and to decrease estrogen receptor levels in MCF-7 breast cancer cells [71], establishing an intersection between cytokine and hormonal signaling in mammary cells. Subsequent studies presented a correlation between IL1α expression, breast cancer severity, and ER-negativity [72,73], but no functional connections have been established.

Due to the expression of endogenous IL1RI in MMECs, we can interrogate whether recombinant IL1α is sufficient to inhibit MMEC growth, rescue 1,25(OH)_2_D_3_-mediated growth inhibition in IL1α knockdown cells, induce p27, and/or induce senescence. However, because intracellular IL1α in epithelial cells likely has a different mode of action from extracellular IL1α [39,59], we have also generated IL1α overexpression vectors with which to infect MMECs and IL1α knock-down MMECs in order to further elucidate the contributions of nuclear, membrane-bound, and secreted IL1α signaling in MMEC survival and proliferation. Whether knocking down IL1RI also attenuates the anti-proliferative effects of 1,25(OH)_2_D_3_ remains to be evaluated. 

As approaches to breast cancer treatment become more complex, the importance of chemoprevention is increasingly evident. While *in vitro* studies have shown that estrogen receptor (ER)-positive breast cancer cell lines are directly growth-inhibited by 1,25(OH)_2_D_3_, ER-negative tumor invasion and angiogenesis are indirectly inhibited by 1,25(OH)_2_D_3_ [52,53]. However, 1,25(OH)_2_D_3_ may be more beneficial in the chemopreventative setting; it is thought to regulate differentiation and maintain mammary gland homeostasis in the presence of mitogenic signals from the microenvironment [53]. If 1,25(OH)_2_D_3_ signaling is lacking or impaired, estrogen-stimulated epithelial proliferation may escape regulatory control. Our study adds to those supporting the relevance of vitamin D_3_ as a chemopreventative agent, and we report a novel mechanistic role for IL1α in the 1,25(OH)_2_D_3_-mediated growth regulation of normal mammary epithelial cells.

## Supporting Information

Figure S1 **Full scans of Western blots for p27, p21 (long exposure), and β actin (short exposure) from Figure 2.** The p21 signal is weak.
(TIF)

Figure S2 **Full scan of Western blot for IL1α and β actin from Figure 4.**
(TIF)

Figure S3 **IL1α knockdown cells are responsive to 1,25(OH)_2_D_3_.** qPCR shows robust induction of Cyp24a1 by 100 nM 1,25(OH)_2_D_3_ (1,25D3) at 24 hrs in MMEC clones (cl.) infected with negative control (NC) and IL1α shRNA.
(TIF)

Figure S4 **1,25(OH)_2_D_3_ induces senescence in the absence of IL1α.** Quantification of senescence-associated beta galactosidase assays revealed that 1,25(OH)_2_D_3_ significantly induced senescence compared to the control treatment (EtOH). Bars labeled “a,” “b,” or “c” are statistically significantly different from each other according to ANOVA and post-hoc Fisher’s LSD test (n = 3 replicates, ~160 cells quantified in each of 10 fields of view per replicate, critical value = 0.05).
(TIF)
