# Supplementary figures and images for: A Role for Interleukin-1 Alpha in the 1,25 Dihydroxyvitamin D3 Response in Mammary Epithelial Cells
Source: PLoS One. 2013 Nov 7;8(11):e81367. doi: 10.1371/journal.pone.0081367 (PMC3820570; doi:10.1371/journal.pone.0081367)

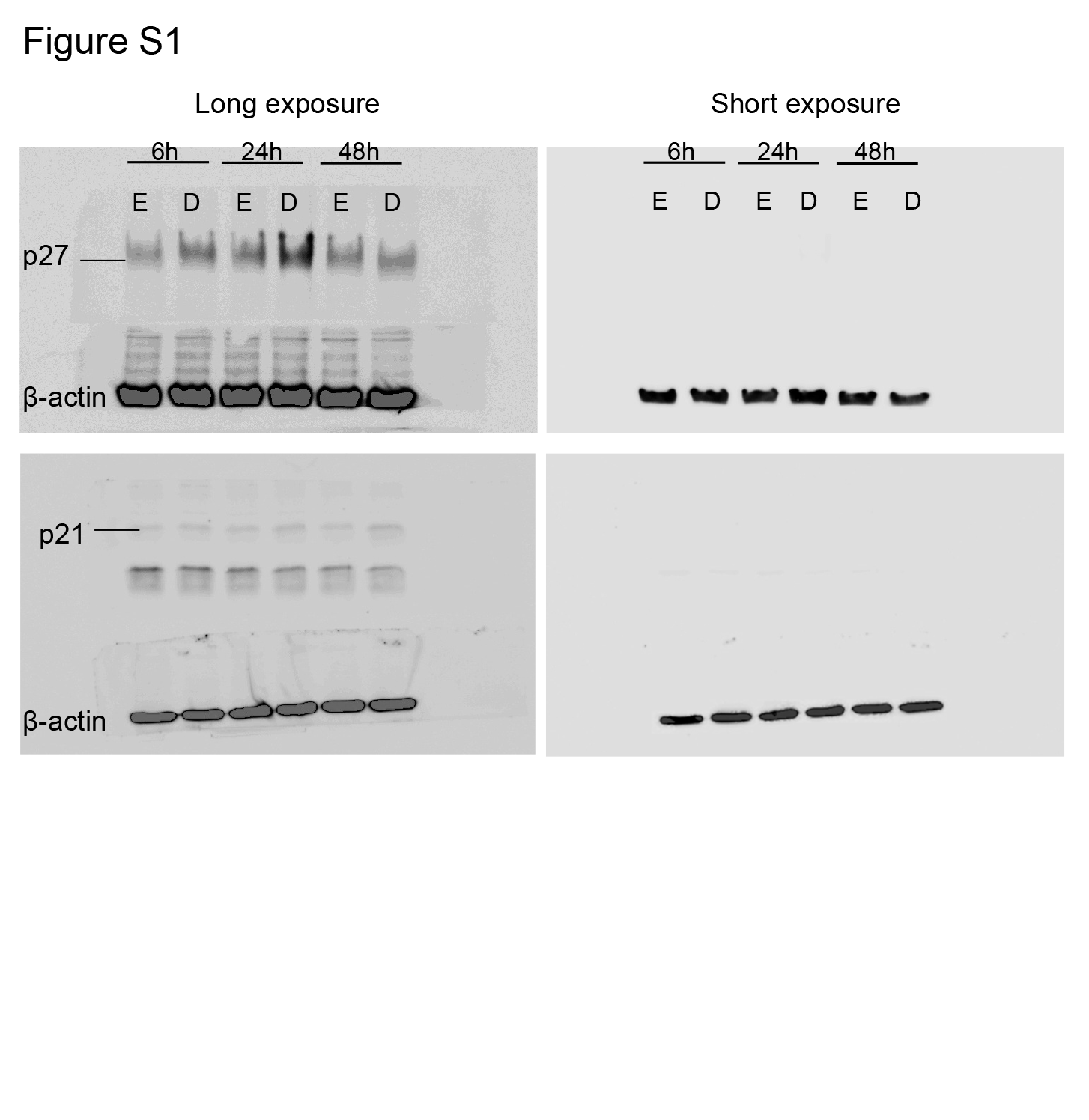

Supplement: Figure S1 — Full scans of Western blots for p27, p21 (long exposure), and β actin (short exposure) from Figure 2. The p21 signal is weak. (TIF) [file pone.0081367.s001.tif]

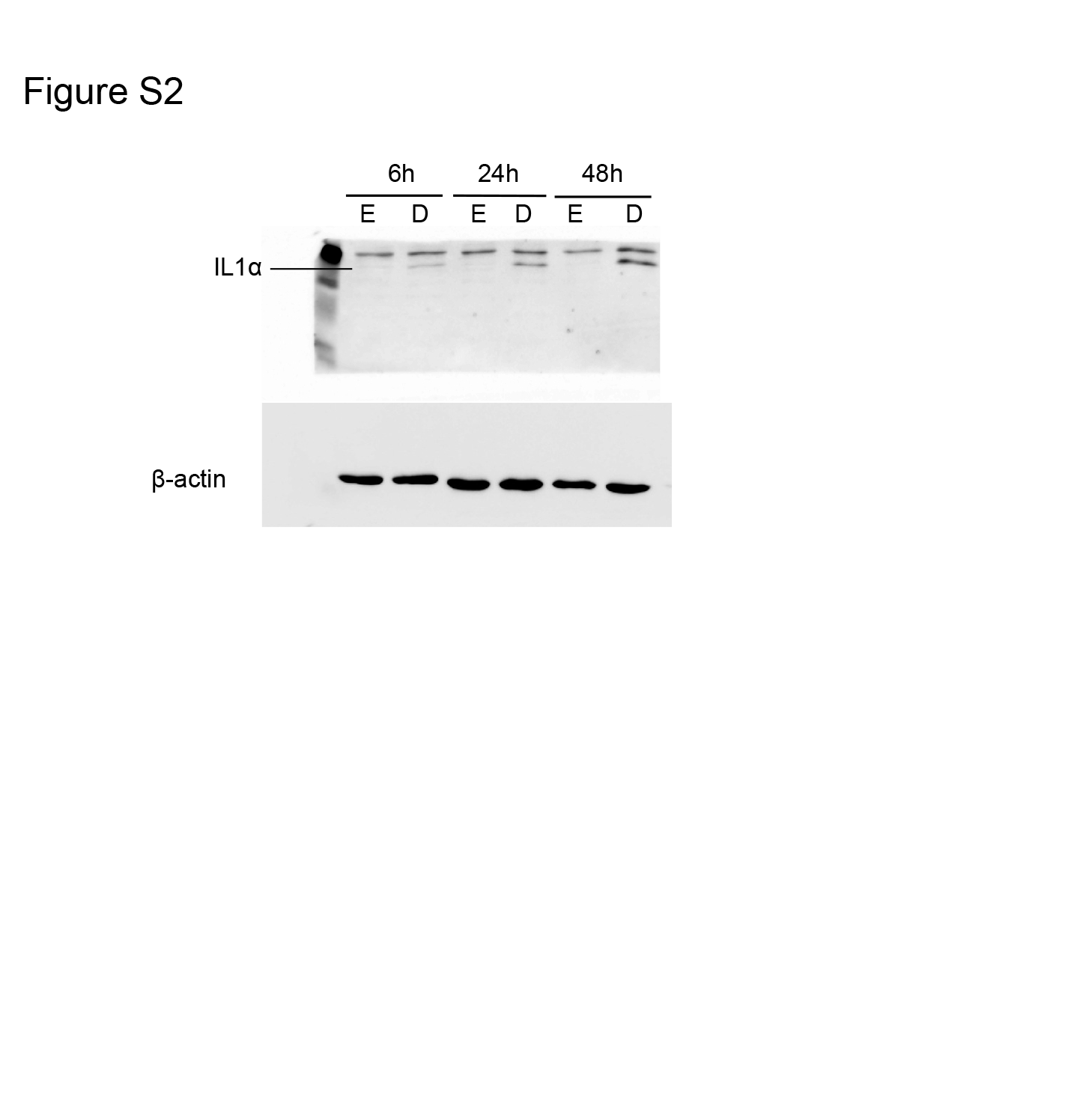

Supplement: Figure S2 — Full scan of Western blot for IL1α and β actin from Figure 4. (TIF) [file pone.0081367.s002.tif]

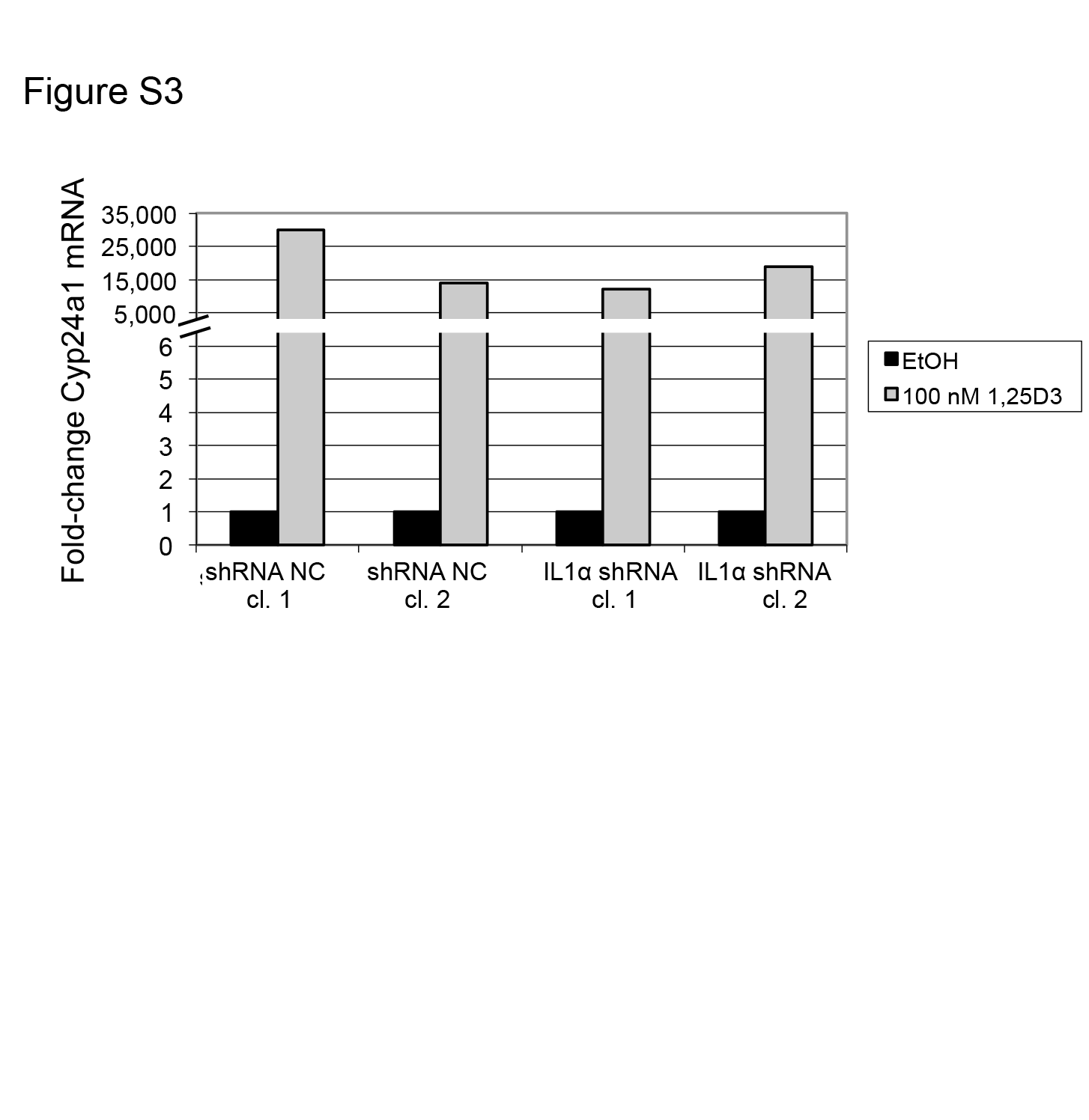

Supplement: Figure S3 — IL1α knockdown cells are responsive to 1,25(OH)2D3. qPCR shows robust induction of Cyp24a1 by 100 nM 1,25(OH)2D3 (1,25D3) at 24 hrs in MMEC clones (cl.) infected with negative control (NC) and IL1α shRNA. (TIF) [file pone.0081367.s003.tif]

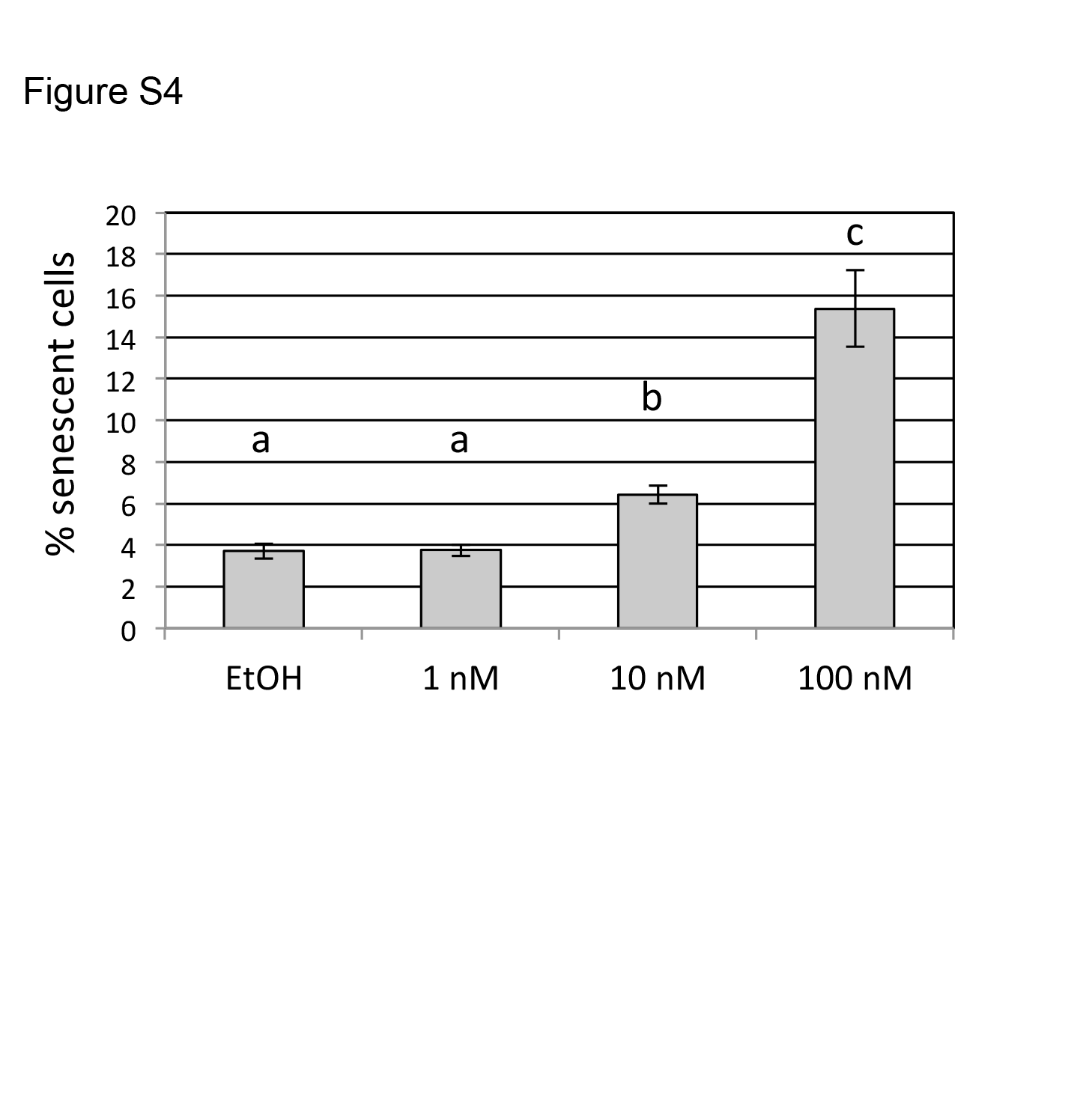

Supplement: Figure S4 — 1,25(OH)2D3 induces senescence in the absence of IL1α. Quantification of senescence-associated beta galactosidase assays revealed that 1,25(OH)2D3 significantly induced senescence compared to the control treatment (EtOH). Bars labeled “a,” “b,” or “c” are statistically significantly different from each other according to ANOVA and post-hoc Fisher’s LSD test (n = 3 replicates, ~160 cells quantified in each of 10 fields of view per replicate, critical value = 0.05). (TIF) [file pone.0081367.s004.tif]
